# Construction and Performance of Novel Oil Catalytic Materials from Electric Arc Furnace Dust

**DOI:** 10.3390/molecules31010035

**Published:** 2025-12-22

**Authors:** Yi-Tong Wang, Kai-Li Dong, Rui Ji, Ya-Jun Wang, Jun-Guo Li, Hang Zhao, Liang-Yi Zhang, Shu-Hao Zhang, Zi-Han Tang, Jie Yang

**Affiliations:** 1 College of Metallurgy and Energy, North China University of Science and Technology, 21 Bohai Street, Tangshan 063210, China; wangyt@ncst.edu.cn (Y.-T.W.); 17739439899@163.com (K.-L.D.); jirui7777@163.com (R.J.); wangyj@ncst.edu.cn (Y.-J.W.); 15530326449@163.com (L.-Y.Z.); psyzhang7@163.com (S.-H.Z.); lisatzh4309@163.com (Z.-H.T.); 2 Yanzhao Iron and Steel Laboratory, North China University of Science and Technology, 21 Bohai Street, Tangshan 063210, China; 3 Qian’an College, North China University of Science and Technology, Qian’an 064400, China

**Keywords:** electric furnace dust, magnetic multiphase catalyst, Na_2_CO_3_, biodiesel, activation mechanism

## Abstract

As a widely sourced solid waste rich in metallic elements such as Fe, Zn, Mn and Ca, electric furnace dust serves as a crucial raw material for preparing catalytic materials. This study employed a three-step process—“acid/alkali modification–impregnation–calcination”—to synthesise an electric furnace dust-based magnetic heterogeneous catalyst for biodiesel production. The catalyst prepared via CH_3_ONa modification combined with Na_2_CO_3_ impregnation achieved stable cycling performance at low temperatures, with 14 cycles yielding a consistent conversion exceeding 93.44 wt%, demonstrating exceptional catalytic activity. The CH_3_ONa modification generates abundant reactive oxygen species on the furnace dust surface, facilitating the binding of hydroxyl oxygen from the active component (Na^+^) to the modified surface (EFD/CH_3_ONa) and thereby anchoring the active species. However, the decline in catalytic performance of the Na_2_CO_3_&(EFD/CH_3_ONa) catalyst after calcination at 600 °C (yield decreasing to 69.77 wt% after 11 stable cycles) was attributed to the detachment and agglomeration of the active component sodium at elevated temperatures. This paper employed electric furnace dust as feedstock to synthesise highly active and stable magnetic multiphase catalysts, thereby not only providing an environmentally sound pathway for industrial solid waste recycling but also offering novel insights for the industrial-scale production of biodiesel.

## 1. Introduction

The swift growth of the global population, the accelerated pace of industrialisation, and the continuous rise in economic standards have intensified the consumption of fossil fuels, precipitating severe issues such as resource depletion, energy shortages, and exorbitant greenhouse gas discharges [1,2]. To mitigate these problems, the identification and development of renewable green energy sources have become particularly crucial. Biodiesel, due to its low greenhouse gas discharges, biodegradability, and renewable attributes, has become a sustainable substitute to fossil fuels [3,4,5]. Biodiesel is usually manufactured as fatty acid methyl ester (FAME) via transesterification of vegetable oils or animal fats with short-chain alcohols in the presence of acid or alkaline catalysts. Currently, owing to its excellent fuel properties, it is widely utilised in diesel engines [6]. However, selecting an appropriate catalyst is pivotal for achieving efficient and cost-effective biodiesel production. Presently, the catalysts applied in the industrial production of biodiesel largely comprise homogeneous and heterogeneous varieties. Whilst homogeneous catalysts (such as NaOH and H_2_SO_4_) offer advantages, including high catalytic activity and mild reaction conditions, these materials are difficult to separate from the products. By comparison, heterogeneous catalysts, on account of their incompatibility with the products, are more readily separable and reusable, making them the preferred catalysts for sustainable biodiesel synthesis [6]. Metal-based solid wastes, due to their wide availability, suitable pore structures, and tunability, are extensively utilised as adsorbents and catalysts in synthetic energy production. However, conventional separation methods (such as centrifugation and filtration) pose significant challenges for catalyst recovery when handling extremely fine-grained heterogeneous catalysts. To minimise losses during heterogeneous catalyst recovery and enhance catalyst stability, magnetic catalysts have emerged as promising materials. Their responsiveness to external magnetic fields enables straightforward and efficient separation with reduced mass loss [7].

Electric furnace dust, as an industrial waste product, contains substantial quantities of Fe, Zn, Mn and Ca oxides alongside minor amounts of free metals. These constituents can catalyse a variety of reactions [8,9], making it a rich source material for the preparation of magnetic catalytic materials. Amdeha et al. [10] pioneered the fabrication of ordered mesoporous silica (MCM-41), nano-zinc oxide (ZnO), and zinc sulphide (ZnS) with blast furnace slag (BFS) and electric furnace dust (EFD) serving as raw materials. These substances were used as composite photocatalysts, exhibiting effective photoreduction activity for highly toxic chromium(VI) and less toxic chromium(III) under ultraviolet irradiation. Wang et al. [11] utilised electric furnace dust as feedstock to prepare an acid–base dual-functional heterogeneous catalyst via sodium carbonate impregnation for catalysing biodiesel production from soybean oil. Under optimised conditions, the initial biodiesel yield reached 99.8 wt%, and after 11 cycles, the yield remained above 90 wt%, demonstrating exceptionally high catalytic activity and stability.

Moreover, electric furnace dust treated through acid leaching, alkali leaching, and similar processes may exhibit favourable carrier properties, such as suitable morphological structure and high specific surface area. The addition of acid enhances the affinity of metal oxides within the dust [12], thereby facilitating the adhesion of active components. The modified dust exhibits an increased specific surface area, generating abundant active sites that enhance the catalytic stability of electric furnace dust [13]. Common alkali-modifying reagents include NaOH, KOH, and NH_3_·H_2_O. Following alkali solution modification, the pore structure and local environment on both the internal and external surfaces of the dust undergo significant alteration [14]. This process generates a large number of oxygen functional groups [12] while introducing alkaline sites [15], thereby further enhancing the catalytic efficiency for biodiesel production. Moreover, alkali modification can effectively reduce ash content and impurities in the dust, thereby increasing the proportion of active components within it. The impregnation method is one of the most commonly employed techniques for preparing supported catalysts. During impregnation, the catalyst support and active components are immersed in a solution. The active components not only adhere to the support surface but also penetrate into the pores of the support via capillary action, thereby forming catalytic materials with high catalytic activity [16]. By adjusting the impregnation temperature, impregnation amount, and impregnation time, the distribution of active components over the catalyst support can be regulated. The more uniform the distribution of these active components, the more conducive it is to enhancing the catalyst’s activity and stability. Catalyst calcination involves high-temperature heating to remove organic matter, moisture, and other impurities from the catalyst. The calcination process influences the catalyst’s surface morphology, structural characteristics, and the dispersion of active sites [17]. Optimising the calcination temperature of materials enables effective modulation of the surface properties of catalysts, thereby further enhancing catalytic activity [18]. To date, no researchers have applied electric furnace dust as a raw material to prepare heterogeneous catalysts that facilitate the production of biodiesel from fats and oils by means of the “acid/alkali modification–impregnation” method.

Hence, this chapter explores the influence of acid–base modification on the catalytic activity of electric furnace dust, with the dust serving as raw material. Employing a combined impregnation–high-temperature calcination method, it examines changes in the chemical composition and structural characteristics of both the original and modified electric furnace dust during the activation process, thereby elucidating the activation mechanism. The final optimised synthetic catalyst exhibits high catalytic activity and satisfactory stability, making it appropriate for catalysing biodiesel production from fats and oils.

## 2. Results and Discussion

### 2.1. Preparation of EFD/S Precursor from Acid/Alkali-Modified Electric Furnace Dust

Characterisation via inductively coupled plasma optical emission spectrometer (ICP-OES) and elemental analysis revealed the following metal element contents in the EFD studied: Fe, Zn, Mn, Ca, Si, Mg, and Na at 38.65, 11.54, 4.78, 4.63, 3.26, 1.74, and 1.71 wt%, respectively. The C, H, and S contents in the EFD raw material were 1.61, 0.43, and 0.59 wt%, respectively.

**Figure 1:**
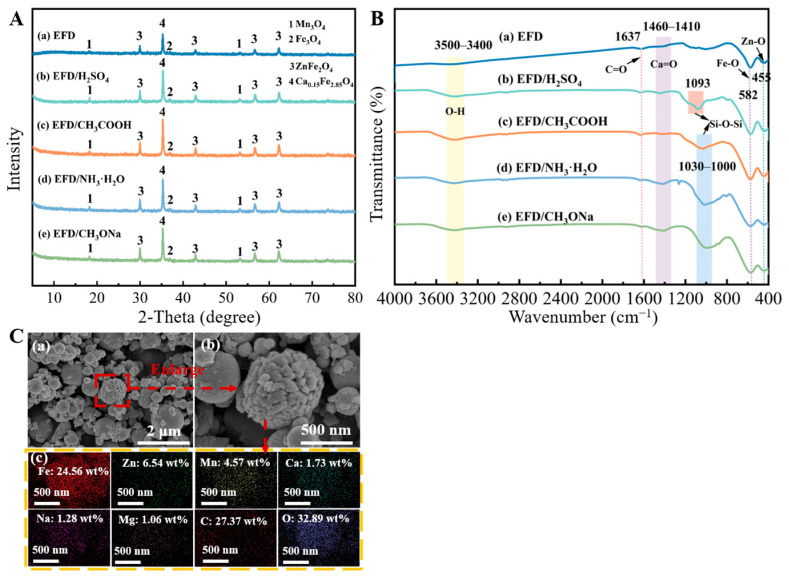
(**A**) XRD patterns and (**B**) FT-IR spectra of (a) EFD, (b,c) acid-modified EFD, and (d–e) alkali-modified EFD; (**C**) SEM-EDS spectrum of EFD/CH_3_ONa. (**C**) (a,b) SEM images and (c) EDS spectrum of EFD/CH_3_ONa.

Under the conditions of a reaction temperature of 65 °C, a reaction time of 2 h, 7 wt% catalyst dosage, and 15/1 alcohol/oil molar ratio, electric furnace dust was directly employed as a catalyst to catalyse soybean oil, and the obtained biodiesel yield was 0.3 wt%. To explore the influence of acid–base modification on the catalytic activity of EFD feedstock, EFD was modified as a catalyst using hydrochloric acid, sulfuric acid, nitric acid, phosphoric acid, sodium hydroxide, potassium hydroxide, ammonia water, acetic acid, oxalic acid, malic acid, citric acid, sodium methoxide, potassium methoxide, and sodium ethoxide, respectively. Under identical conditions, the biodiesel yield from the catalysis of soybean oil was merely 0.1~3.6 wt%. To investigate the effects of acid (or alkali) modification on the chemical composition and structural properties of EFD, characterisation analyses were conducted on both unmodified and modified EFD using X-ray diffractometer (XRD), field-emission scanning electron microscope equipped with an energy-dispersive spectrometer (SEM-EDS), Fourier transform infrared spectrometer (FT-IR), X-ray photoelectron spectrometer (XPS), Brunauer–Emmett–Teller (BET), thermogravimetric analyser (TG), and vibrating sample magnetometer (VSM). The EFD raw material comprises mineral phases such as Mn_3_O_4_, Fe_3_O_4_, ZnFe_2_O_4_ and Ca_0.15_Fe_2.85_O_4_ (Figure 1A(a)). The spinel-structured ZnFe_2_O_4_ [19] exhibited high-intensity diffraction peaks at multiple angles, including 2θ = 29.8°, 42.9°, 56.7° and 62.3°, indicating that Fe and Zn in the EFD raw material predominantly existed in the form of ZnFe_2_O_4_. Mn_3_O_4_ appeared at 2θ = 18.3° and 53.4°, while Ca_0.15_Fe_2.85_O_4_ appears at 2θ = 35.4°. Additionally, a low-intensity diffraction peak corresponding to the Fe_3_O_4_ (311) crystal plane was detected at 2θ = 35.4° [20], which also explained the magnetic properties exhibited by the electric furnace dust. Following modification of EFD with H_2_SO_4_, CH_3_COOH, NH_3_·H_2_O and CH_3_ONa, no new mineral phases were detected. However, it was evident that following acid (or alkali) modification, the intensities of the ZnFe_2_O_4_ and Ca_0.15_Fe_2.85_O_4_ diffraction peaks in the EFD increased significantly (Figure 1A(b–e). This indicated that after acid or alkali treatment, the amorphous oxides or weakly crystalline impurities on the surface of the electric furnace dust were dissolved, resulting in a reduced proportion of amorphous phase components. SEM and EDS analysis of CH_3_ONa-modified EFD (EFD/CH_3_ONa) is shown in Figure 1C(a–c). The EFD/CH_3_ONa sample primarily consisted of irregular spherical particles ranging from 50 to 600 nm in size. Some submicron particles had agglomerated due to electrostatic interactions, forming larger spherical bodies and clusters [21]. Individual spherical particles exhibited a loose, porous surface, likely resulting from reactions between the alkaline solution and metallic dust during the modification process [22]. EDS analysis (Figure 1C(c)) revealed the mass ratios of Na/Fe, Na/Zn, Fe/Mn, Ca/Fe, and Mg/Fe on the surface of the EFD/CH_3_ONa sample to be 1/19.19 (Na at 1.28 wt% and Fe at 24.56 wt%), 1/5.11 (Zn at 6.54 wt%), 1/0.19 (Mn at 4.57 wt%), 1/14.20 (Ca at 1.73 wt%), and 1/23.17 (Mg at 1.06 wt%). These elements were uniformly distributed throughout the entire detection range.

Broad peak bands between 3500 cm^−1^ and 3400 cm^−1^ were observed in the FT-IR spectra of both EFD and modified EFD (Figure 1B(a–e)), attributed to the stretching vibrations of O-H groups in water molecules [23]. Bending vibration peaks characteristic of Fe-O [24] and Zn-O functional groups were identified near 582 cm^−1^ and 455 cm^−1^, respectively, consistent with XRD analysis. The characteristic peak near 1637 cm^−1^ corresponded to the C=O group in the carbonyl [25], suggesting the existence of residual organic matter in the EFD. Following acid (or base) modification of the EFD, the resulting EFD/H_2_SO_4_, EFD/CH_3_COOH, EFD/NH_3_·H_2_O, and EFD/CH_3_ONa catalysts exhibited Si-O-Si vibrational peaks near 1090 cm^−1^, 1028 cm^−1^, 1018 cm^−1^, and 1003 cm^−1^, respectively [26]. This phenomenon was likely attributable to the acid (or base) solution treatment disrupting the external Fe, Zn, and Ca compounds, thereby exposing the internal silicon oxides [27].

**Figure 2:**
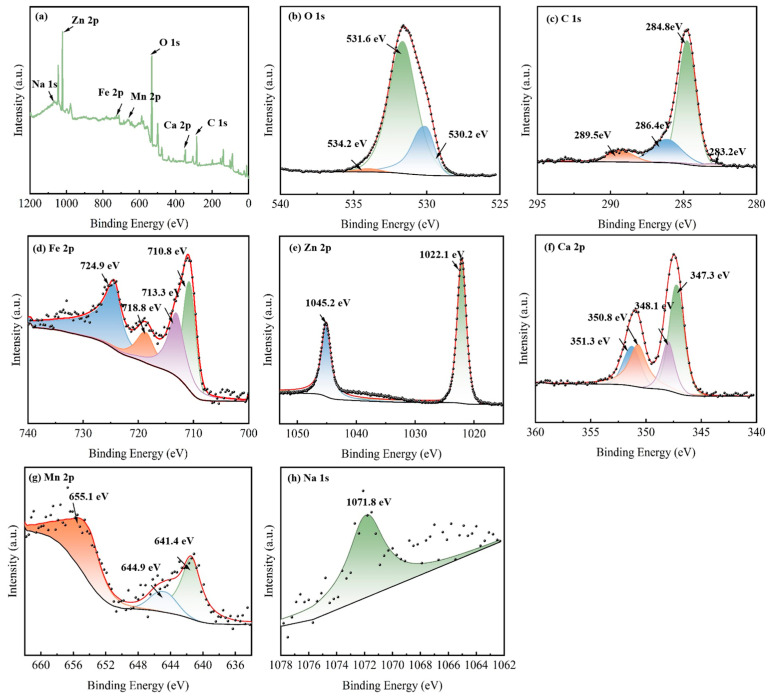
XPS spectra of EFD/CH_3_ONa: (**a**) total spectrum, (**b**) O 1s, (**c**) C 1s, (**d**) Fe 2p, (**e**) Zn 2p, (**f**) Ca 2p, (**g**) Mn 2p, and (**h**) Na 1s.

To gain further insight into element composition and chemical states on the surface of modified EFD as a catalytic material, full-spectrum and fine-spectrum XPS analyses were conducted on the EFD/CH_3_ONa sample. All binding energy data were charge-corrected against the standard peak at 284.8 eV for C 1s. In the full XPS spectrum (Figure 2a), EFD/CH_3_ONa was primarily composed of Na 1s, Zn 2p, Fe 2p, Mn 2p, O 1s, Ca 2p, and C 1s peaks. As shown in Figure 2b, the O 1s spectrum exhibited three distinct peaks with binding energies of 530.2 eV, 531.6 eV, and 534.2 eV, corresponding to lattice oxygen (O_L_), O^2−^ in oxygen vacancies (O_V_), and surface hydroxyl oxygen (-OOH), respectively. These lattice oxygen and oxygen vacancy sites constituted the critical active constituents enhancing catalytic performance and electron transport capacity [28]. The C1s spectrum exhibited three distinct peaks with binding energies of 284.8 eV, 286.4 eV, and 289.5 eV (Figure 2c), associated with the C-C/C=C [29], C-O [30], and C=O [29] functional groups, respectively, in EFD/CH_3_ONa. In Figure 2d, the Fe 2p peaks near 710.8 eV, 713.3 eV, and 724.9 eV correspond to Fe-C/Fe, Fe^3+^, and Fe^2+^, respectively, indicating the simultaneous presence of Fe_3_O_4_ and Fe_2_O_3_ in the sample. A satellite peak of Fe 2p appeared at a binding energy of 718.8 eV [31], confirming the presence of Fe^3+^ or Fe^2+^ oxidation states in the sample. The high-resolution spectrum of the Zn 2p (Figure 2e) displayed two characteristic peaks at 1022.1 eV and 1045.2 eV, matching the spin–orbit peaks of ZnO’s Zn 2p_3/2_ and Zn 2p_1/2_, respectively [32]. For EFD, due to spin–orbit coupling, the Ca 2p region showed two peaks at 348.6 eV and 350.8 eV, matching Ca 2p_1/2_ and Ca 2p_3/2_, respectively, as shown in Figure 2f, both indicating the presence of calcium in the material as Ca^2+^ [30]. As depicted in Figure 2g, in the high-resolution Mn 2p XPS spectrum for EFD/CH_3_ONa, a sharp peak was observed at 641.4 eV, assigned to Mn 2p_3_/_2_. Deconvolution of this peak uncovered two separate peaks at 641.4 and 644.9 eV, which correspond to Mn^2+^ and Mn^3+^, respectively [33]. A characteristic peak at 1071.8 eV was fitted for Na 1s, indicating the presence of Na^+^ in the sample [34].

**Figure 3:**
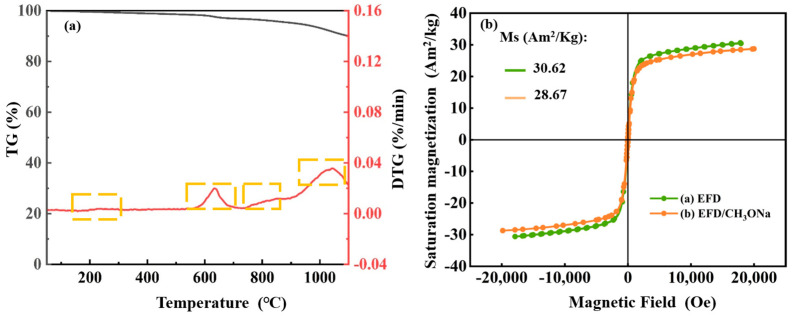
(**a**) TG-DTG curves of EFD/CH_3_ONa and (**b**) VSM curves of EFD and EFD/CH_3_ONa.

To explore the thermal stability of the EFD/CH_3_ONa material, TG-DTG analysis was conducted over the range of temperatures for 0–1100 °C, with results presented in Figure 3a. The entire heating process could be subdivided into four distinct stages of mass loss. During the first stage, with the temperature increasing from ambient temperature to 300 °C, a slight decrease in mass was observed for EFD/CH_3_ONa. This was primarily attributed to the removal of entrapped free water within the sample [35]. In the second stage, as the temperature rose to the 600–700 °C range, a marked acceleration in weight loss occurred (1.48 wt%), attributable to mass loss stemming from the vaporisation of chlorides in EFD/CH_3_ONa or the decomposition of organic matter (confirmed by FT-IR) [36]. In the third stage, as temperature continued to rise, a gradual weight loss occurred between 750 and 900 °C. This could be attributed to the carbothermal reduction of metal oxides within the sample [37], releasing gases such as carbon monoxide. The fourth stage, occurring when temperatures exceeded 1000 °C, saw the sample lose approximately 3.05 wt% of its mass. This phase likely represented the decomposition of Ca_0.15_Fe_2.85_O_4_ (consistent with XRD results) at elevated temperatures, releasing minor quantities of oxygen. Additionally, the magnetic characteristics of the catalytic material constituted a significant factor influencing catalyst stability. The VSM results are presented in Figure 3b. The hysteresis loops of the samples measured before and after CH_3_ONa modification exhibited symmetrical “S”-shaped curves, demonstrating superparamagnetic characteristics [38]. The saturation magnetisation of EFD and EFD/CH_3_ONa was 30.62 Am^2^/kg and 28.67 Am^2^/kg, respectively. The modified EFD exhibited a slight decrease in magnetic properties, potentially attributable to alterations in the relative content of internal Fe_3_O_4_ following CH_3_ONa modification. For instance, Fe_3_O_4_ might undergo hydrolysis within the alkaline environment of CH_3_ONa, yielding weakly magnetic iron hydroxide (FeOOH) or non-magnetic ferrites.

### 2.2. Preparation of M&(EFD/S) Catalyst by Metal Salt Impregnation Activation of EFD/S

The modified samples did not exhibit enhanced catalytic activity compared to the raw EFD material. However, following modification, the EFD/S samples demonstrated varying degrees of improvement in both specific surface area and the chemical environment of surface elements, suggesting potential as a catalyst precursor. Therefore, this section employed EFD as a precursor both before and after acid (or base) modification. Using metal salts as active agents, the active substances were loaded onto the modified and unmodified EFD via impregnation. The influence of metal salt type on catalytic activity was subsequently investigated.

Select CdCO_3_, BaCO_3_, Li_2_CO_3_, Na_2_CO_3_·10H_2_O, NaHCO_3_, K_2_CO_3_, CH_3_ONa, C_2_H_5_ONa, CH_3_OK, Na_2_CO_3_, and other activators to treat electric furnace dust. Under the conditions of 65 °C reaction temperature, 2 h reaction time, 7 wt% catalyst dosage, and 15/1 alcohol/oil molar ratio, soybean oil was catalysed to produce biodiesel; the resulting yields exhibited significant variation when catalysing the conversion of soybean oil into biodiesel (Table 1). The first-cycle biodiesel yields catalysed by CdCO_3_, BaCO_3_, and Li_2_CO_3_-impregnated electric furnace dust were 0.8, 0.7, and 4.2 wt%, respectively. Compared with the original electric furnace dust (with an initial catalytic yield of 0.3 wt%), the catalytic activity did not show a significant improvement. Catalysts prepared using the same method—Na_2_CO_3_·10H_2_O&EFD, NaHCO_3_&EFD, K_2_CO_3_&EFD, CH_3_ONa&EFD, C_2_H_5_ONa&EFD, CH_3_OK&EFD, and Na_2_CO_3_&EFD—exhibited substantially enhanced catalytic activity. Their first-cycle yields reached 66.6 wt%, 88.0 wt%, 92.7 wt%, 89.8 wt%, 84.3 wt%, 94.4 wt%, and 85.6 wt%, respectively. However, their cycling stability proved inadequate, with only Na_2_CO_3_&EFD maintaining a yield of 88.6 wt% after the second cycle. It should also be noted that the biodiesel yield increased following the second catalytic cycle. This may be attributed to the activation of active sites on the catalyst surface through interaction with the substrate (methanol or fatty oils), leading to the formation of more stable active centres. This phenomenon was consistent with the findings reported by Wang et al. [11]. Consequently, Na_2_CO_3_ was chosen as the optimal activator for the subsequent optimisation of activity.

A series of Na_2_CO_3_&(EFD/S) catalyst systems was prepared using acid (or base)-modified EFD as the precursor, activated via Na_2_CO_3_ impregnation. The study investigated the combined effect of different acid (or base) modifiers with Na_2_CO_3_ impregnation on catalyst activity and stability. Under identical catalytic conditions, the yields from soybean oil catalysis are presented in Table 2. The catalytic performance of acid (or base)-modified EFD exhibited a more pronounced enhancement following impregnation. During the initial cycle, all modified EFD achieved a biodiesel yield of 100.0 wt%. Cycling experiments revealed that catalysts such as Na_2_CO_3_&(EFD/HCl), Na_2_CO_3_&(EFD/HNO_3_), Na_2_CO_3_&(EFD/NaOH), Na_2_CO_3_&(EFD/H_2_C_2_O_4_), and Na_2_CO_3_&(EFD/C_2_H_5_ONa) catalysts maintained relatively high biodiesel yields throughout the cycles. However, by the seventh cycle, yields abruptly declined to 49.4 wt%, 75.1 wt%, 53.7 wt%, 51.5 wt% and 12.1 wt%, respectively. The biodiesel yields catalysed by Na_2_CO_3_&(EFD/H_2_SO_4_), Na_2_CO_3_&(EFD/H_3_PO_4_), Na_2_CO_3_&(EFD/KOH), Na_2_CO_3_&(EFD/NH_3_·H_2_O), Na_2_CO_3_&(EFD/CH_3_COOH), Na_2_CO_3_&(EFD/C_4_H_6_O_5_) and Na_2_CO_3_&(EFD/C_6_H_8_O_7_) decreased to 65.8 wt%, 70.2 wt%, 33.2 wt%, 66.0 wt%, 60.0 wt%, 50.7 wt%, and 60.1 wt% in the 8th, 9th, 10th, 10th, 11th, 9th, and 9th cycles of reuse, respectively, exhibiting excellent catalytic stability. More surprisingly, catalysts prepared by impregnating sodium carbonate with a combination of sodium methoxide and potassium methoxide modifications—namely Na_2_CO_3_&(EFD/CH_3_ONa) and Na_2_CO_3_&(EFD/CH_3_OK)—achieved yields exceeding 90.0 wt% across twelve cycling tests. The former maintained a yield of 93.4 wt% even after 14 catalytic cycles, demonstrating optimal cycling stability while retaining high catalytic activity.

To investigate the changes in chemical composition and structural characteristics of EFD and EFD/CH_3_ONa during the activation process and elucidate the activation mechanism, the samples underwent detailed characterisation analysis employing a combination of XRD, SEM-EDS, BET, XPS, FT-IR, and VSM techniques.

**Figure 4:**
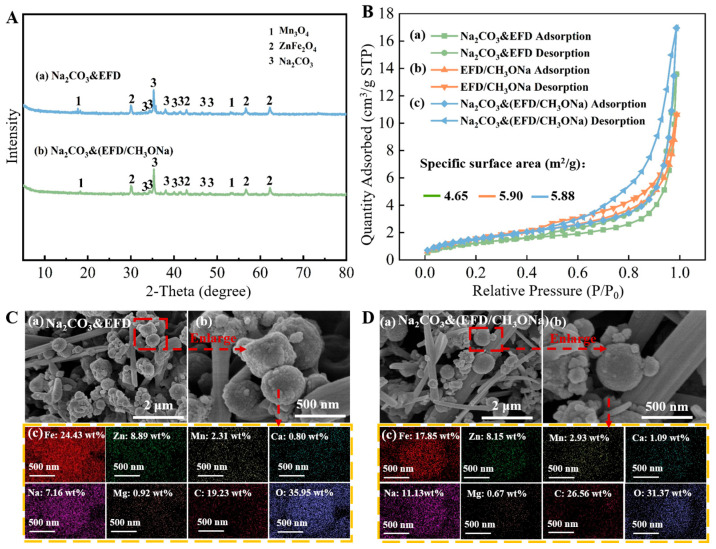
(**A**) XRD patterns of (a) Na_2_CO_3_&EFD and (b) Na_2_CO_3_&(EFD/CH_3_ONa); (**B**) BET analysis of (a) Na_2_CO_3_&EFD, (b) EFD/CH_3_ONa, and (c) Na_2_CO_3_&(EFD/CH_3_ONa); SEM-EDS spectrum of (**C**) Na_2_CO_3_&EFD and (**D**) Na_2_CO_3_&(EFD/CH_3_ONa). (**C**) (a,b) SEM images and (c) EDS spectrum of Na_2_CO_3_&EFD; (**D**) (a,b) SEM images and (c) EDS spectrum of Na_2_CO_3_&(EFD/CH_3_ONa).

Figure 4A(a,b) displays the XRD patterns of the Na_2_CO_3_&EFD and Na_2_CO_3_&(EFD/CH_3_ONa) catalysts, investigating changes in their crystalline compositions. As observed in Figure 4A(a), following Na_2_CO_3_ impregnation, the sample exhibited Na_2_CO_3_ diffraction peaks at 2θ = 33.4°, 34.5°, 35.3°, 37.9°, 39.9°, 41.5°, 46.4°, and 48.4° [39,40]. Additionally, the original Ca_0.15_Fe_2.85_O_4_ crystalline phase in EFD disappeared without the formation of any new substances, indicating that Na_2_CO_3_ may have adsorbed onto the EFD surface in crystalline form. Further analysis using the BET method examined changes in the specific surface area and pore structure of EFD and EFD/CH_3_ONa before and after Na_2_CO_3_ impregnation (Figure 4B). Since the specific surface area, pore volume, and average pore diameter of EFD were 4.19 m^2^/g, 0.0009 cm^3^/g, and 2.02 nm, respectively, after modification with sodium methoxide, the specific surface area, pore volume, and average pore diameter of the dust all increased to a certain extent, rising to 5.90 m^2^/g, 0.016 cm^3^/g, and 11.13 nm, respectively. This indicated that alkaline modification was an efficient approach to enhancing the specific surface area of electric furnace dust, which was beneficial for EFD to act as a carrier for adsorbing active components. The catalyst Na_2_CO_3_&(EFD/CH_3_ONa) obtained after impregnation of EFD/CH_3_ONa with Na_2_CO_3_ exhibited a further decrease in specific surface area to 5.88 m^2^/g, indicating that the internal pores of EFD/CH_3_ONa were filled by Na_2_CO_3_. SEM-EDS analysis of the catalyst’s microstructure generated the results presented in Figure 4C,D. The surface of Na_2_CO_3_&EFD (Figure 4C(a,b)) comprised an interwoven structure of smooth rod-like features (200~500 nm) and loosely porous spherical particles (20~500 nm), exhibiting an overall well-ordered floral cluster arrangement. This morphology facilitated contact between reactant molecules and active sites [33]. As no rod-like morphology was present in the EFD sample prior to Na_2_CO_3_ impregnation, the rod-like structures observed may represent Na_2_CO_3_ crystallites. The loose spherical particles on the surface likely resulted from Na attachment to the catalyst surface during Na_2_CO_3_ dissolution in the impregnation process, causing the content of Na content to increase from 1.71 wt% (EFD) to 7.16 wt% (Na_2_CO_3_&EFD). The morphology of Na_2_CO_3_&(EFD/CH_3_ONa) (Figure 4D(a,b)) resembled that of Na_2_CO_3_&EFD, but exhibited a greater number of fine rod-like structures. This resulted in a more porous overall structure, enhancing the diffusion efficiency of reactants across the catalyst surface and thereby promoting reaction rate improvement [41]. This explained why EFD/CH_3_ONa, as a precursor, facilitated more favourable sodium loading compared to EFD. Furthermore, the EDS patterns of Na_2_CO_3_&EFD and Na_2_CO_3_&(EFD/CH_3_ONa) (Figure 4C(c),D(c)) revealed that the primary metallic elements on the catalyst surface were Fe, Zn, Na, and Mn, uniformly dispersed throughout the catalyst. Notably, the Na content in the latter increased by 55.45%, consistent with BET analysis results. Additionally, the mass ratios of Na/Fe, Na/Zn, Fe/Mn, Ca/Fe, and Mg/Fe on the Na_2_CO_3_&(EFD/CH_3_ONa) surface were 1/1.60 (Na at 11.13 wt% and Fe at 17.85 wt%), 1/0.73 (Zn at 8.15 wt%), 1/0.16 (Mn at 2.93 wt%), 1/16.38 (Ca at 1.09 wt%), and 1/26.64 (Mg at 0.67 wt%). Notably, the Na/Fe mass ratio represented a substantial increase from the original EFD/CH_3_ONa value of 1/19.19, confirming the successful loading of Na_2_CO_3_.

**Figure 5:**
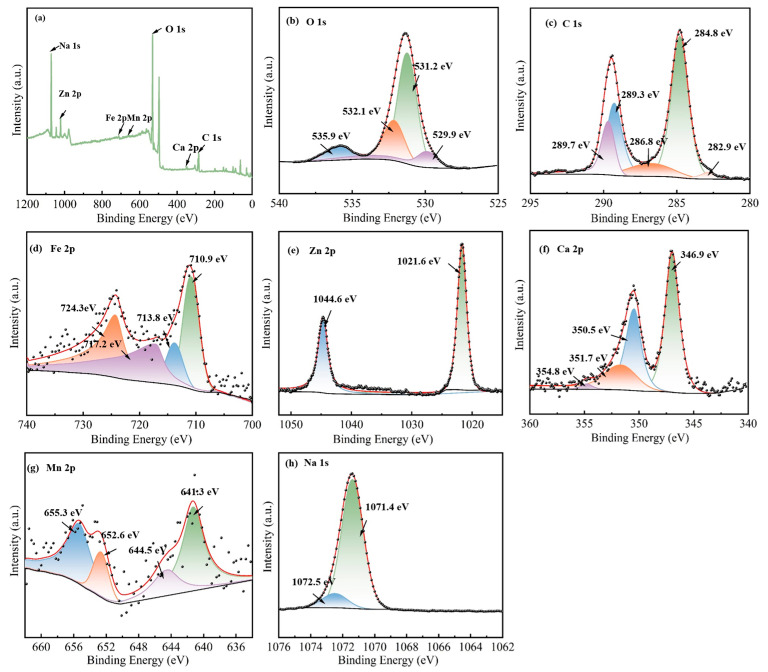
XPS spectra of Na_2_CO_3_&(EFD/CH_3_ONa): (**a**) total spectrum, (**b**) O 1s, (**c**) C 1s, (**d**) Fe 2p, (**e**) Zn 2p, (**f**) Ca 2p, (**g**) Mn 2p, and (**h**) Na 1s.

XPS analysis was utilised to explore the activation mechanism of the Na_2_CO_3_&(EFD/CH_3_ONa) catalyst, with relevant findings summarised in Figure 5. As demonstrated by the broad XPS spectrum in Figure 5a, the presence of Na, Zn, Fe, Mn, O, Ca, and C elements on the catalyst surface. The atomic percentage of Na increased from 0.53% (EFD/CH_3_ONa) to 16.28% (Na_2_CO_3_&(EFD/CH_3_ONa)), indicating successful impregnation of Na onto the EFD/CH_3_ONa precursor. The characteristic peaks of O 1s (Figure 5b) detected lattice-site oxygen (O_L_) at 529.85 eV, 531.21 eV, and 532.12 eV, respectively [38], adsorbed oxygen (Os), and surface hydroxyl oxygen (-OOH), with a higher proportion of surface hydroxyl oxygen (-OOH) (compared to EFD/CH_3_ONa), indicating the generation of more oxygen vacancies in the catalyst [42]. Furthermore, compared to the O 1s spectrum of EFD/CH_3_ONa, a new peak at 535.9 eV corresponded to Na-OH [43], potentially representing a key active site for catalyst activity. Figure 5c displays the split peak spectrum of C 1s, wherein the three observed peaks at 284.78 eV, 286.78 eV, and 289.27 eV were assigned to C-C/C=C, C-O, and C=O in Na_2_CO_3_·(EFD/CH_3_ONa), respectively. In Figure 5d, Fe 2p peaks at 710.93 eV, 713.81 eV, and 724.30 eV were detected, corresponding to Fe-C/Fe, Fe^3+^, and Fe^2+^, respectively. A satellite peak of Fe 2p was observed at a binding energy of 717.15 eV, showing little variation compared to EFD/CH_3_ONa. The high-resolution Zn 2p spectrum (Figure 5e) exhibited two characteristic peaks were observed at 1021.64 eV and 1044.61 eV, which are attributed to the spin–orbit split peaks of the Zn 2p_3/2_ and Zn 2p_1/2_ orbitals in ZnO, respectively. Comparing this with the narrow-scan Zn 2p spectrum corresponding to EFD/CH_3_ONa (Figure 2e), it was evident that the binding energy positions had shifted at both sites. This indicated that the introduction of Na_2_CO_3_ had changed the local chemical environment of Zn present in the electric furnace dust. The Ca 2p spectrum revealed two peaks, Ca 2p_1/2_ and Ca 2p_3/2_ (Figure 5f), both indicating that Ca in Na_2_CO_3_&(EFD/CH_3_ONa) remained in the Ca^2+^ form. As depicted in Figure 5g, in the X-ray photoelectron spectroscopy (XPS) spectrum of Na_2_CO_3_&(EFD/CH_3_ONa), the high-resolution Mn 2p region exhibited two distinct peaks at approximately 642.3 eV and 654 eV. These peaks correspond to the spin–orbit-split Mn 2p_1/2_ and Mn 2p_3/2_ peaks, respectively. Furthermore, the Mn 2p_1/2_ peak was resolved into two separate, distinct sub-peaks, attributed to Mn^2+^ (641.3 eV) and Mn^4+^ (644.5 eV). Similarly, the Mn 2p_3/2_ peak was decomposed into two peaks centred at 652.6 and 655.3 eV, corresponding to Mn^2+^ and Mn^3+^, respectively. A single main peak at 1071.4 eV was fitted for Na 1s, representing the Na^+^ present in the sample [35].

**Figure 6:**
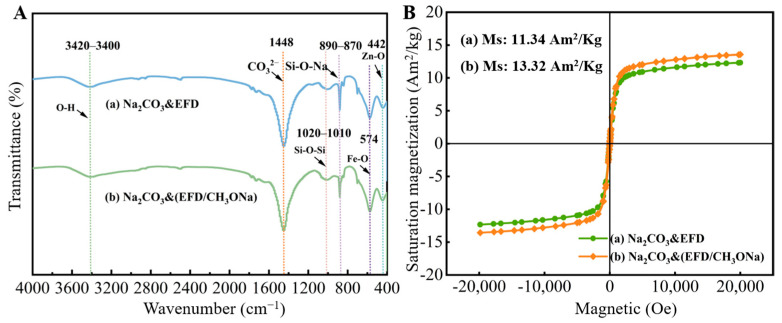
(**A**) FT-IR spectrum and (**B**) VSM diagram of (a) Na_2_CO_3_&EFD and (b) Na_2_CO_3_&(EFD/CH_3_ONa).

FT-IR spectra, shown in Figure 6A(a,b), were used to analyse the functional group composition of the Na_2_CO_3_&EFD and Na_2_CO_3_&(EFD/CH_3_ONa) catalysts. A broad band between 3420 cm^−1^ and 3400 cm^−1^ was observed in both sets of samples, attributed to the stretching vibration of the O-H group in water molecules. Fe-O and Zn-O functional groups were observed near 574 cm^−1^ and 442 cm^−1^, respectively, consistent with the crystalline phases present in the original electric furnace dust. The intense characteristic peak near 1448 cm^−1^ corresponded to the CO_3_^2−^ ion in sodium carbonate. Additionally, a peak at 887 cm^−1^ was analysed, representing the Si-O-Na functional group [44,45]. This confirmed the successful anchoring of Na^+^ onto the surface of EFD/CH_3_ONa precursors. This finding was consistent with the XPS characterisation of EFD/CH_3_ONa. Figure 6B analyses the magnetic susceptibility of the Na_2_CO_3_&EFD and Na_2_CO_3_&(EFD/CH_3_ONa) catalysts. It was evident that after Na_2_CO_3_ impregnation, the saturation magnetisation of both EFD and EFD/CH_3_ONa decreased to 12.31 Am^2^/Kg and 13.57 Am^2^/Kg, respectively. Nevertheless, they retained superparamagnetic characteristics, indicating that after participating in the transesterification reaction, the catalyst could be separated from reactants using an external magnet, facilitating its reuse.

Calcination can stabilise the catalyst structure. Consequently, the following subsection will investigate whether different calcination temperatures prove beneficial for enhancing the catalytic performance of Na_2_CO_3_&(EFD/CH_3_ONa).

### 2.3. Effect of High-Temperature Calcination on the Catalytic Performance of M&(EFD/S)

The calcination temperature significantly influences catalyst performance; selecting an appropriate temperature aids in stabilising the catalyst structure while enhancing the catalytic material’s activity and stability. Herein, the optimal catalyst Na_2_CO_3_&(EFD/CH_3_ONa) identified in the preceding section is subjected to calcination at temperatures ranging from 300 to 900 °C. This study explored the influence of varied temperatures on the catalytic performance of the Na_2_CO_3_&(EFD/CH_3_ONa) catalyst. The cyclic test yields of biodiesel produced from soybean oil using the catalyst (Na_2_CO_3_&(EFD/CH_3_ONa))_T_ are presented in Table 3. At a catalyst loading of 7 wt%, an alcohol/oil molar ratio of 15/1, and a reaction temperature of 65 °C for 2 h, the initial biodiesel yields were 98.8 wt% (300 °C), 99.7 wt% (400 °C), 99.9 wt% (500 °C), 99.8 wt% (600 °C), 99.8 wt% (700 °C), 99.9 wt% (800 °C), and 99.7 wt% (900 °C). It was evident that all catalysts exhibited high activity following calcination treatment. After cyclic testing, only the (Na_2_CO_3_&(EFD/CH_3_ONa))_600_ samples demonstrated superior stability, sustaining 11 stable catalytic cycles with biodiesel yields consistently above 90.0 wt%. Furthermore, it was observed that the (Na_2_CO_3_&(EFD/CH_3_ONa))_600_ sample exhibited the slowest decline in activity during the 12th catalytic cycle compared to other samples. However, its stability showed a significant reduction when compared to the uncalcined sample (Na_2_CO_3_&(EFD/CH_3_ONa)). This decline in stability was hypothesised to be associated with the detachment or agglomeration of active components on the catalyst surface caused by high-temperature calcination.

**Figure 7:**
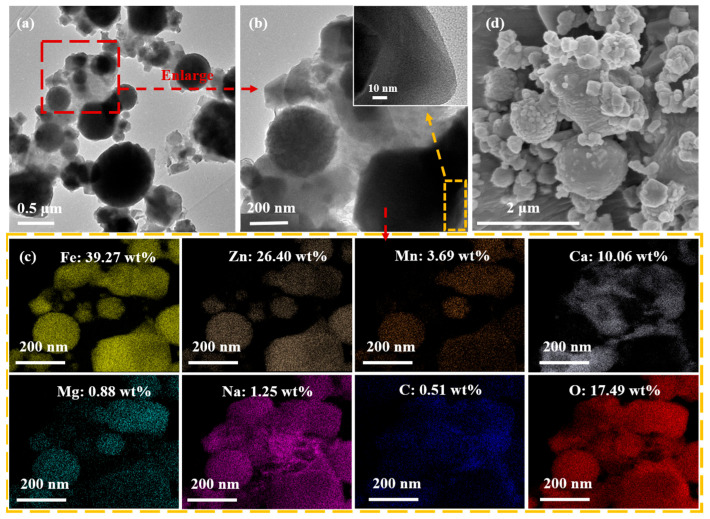
(**a**–**c**) TEM-EDS spectra and (**d**) SEM images of (Na_2_CO_3_&(EFD/CH_3_ONa))_600_.

To verify the above conjecture, characterisation analysis of (Na_2_CO_3_&(EFD/CH_3_ONa))_600_ was conducted using high-resolution transmission electron microscope–energy-dispersive spectrometer (HRTEM-EDS), SEM, XPS, XRD, FT-IR, chemical adsorption analyser (CO_2_-TPD), and TG. As shown in Figure 7a–d, the morphology of Na_2_CO_3_&(EFD/CH_3_ONa) underwent significant alteration following high-temperature calcination. As shown in Figure 4D(a,b), the Na_2_CO_3_&(EFD/CH_3_ONa) powder comprised numerous fine rod-like structures and loose, porous spherical particles. Following calcination at 600 °C, the rod-like structures disappeared, and agglomeration occurred on the surfaces of the spherical particles. As spherical agglomerates proliferate, they obstruct the catalyst’s porous structure and block pores that could accommodate active sites, thereby reducing the diffusion efficiency of reactants between active sites [46]. This phenomenon was one cause of the decline in catalyst activity following calcination. Combined with the scanning energy spectrum of (Na_2_CO_3_&(EFD/CH_3_ONa))_600_ (Figure 7c), the relative contents of Fe, Zn, Mn, Ca, Mg, Na, C, and O after calcination at 600 °C were 39.27, 26.40, 3.69, 10.06, 0.88, 1.25, 0.51, and 17.49 wt%, respectively. The corresponding mass ratios for Na/Fe, Na/Zn, Fe/Mn, Ca/Fe and Mg/Fe were 1:31.42, 1:21.12, 1:0.09, 1:3.90 and 1:44.63, respectively. Compared to Na_2_CO_3_&(EFD/CH_3_ONa), the contents of Mn and Mg showed little change, while those of Fe, Zn, and Ca increased. This might stem from calcination at high temperatures, promoting the transformation of crystalline phases towards ZnFe_2_O_4_ and Ca_0.15_Fe_2.85_O_4_. Additionally, substantial decreases in Na, C, and O contents were observed. This might result from the reaction of crystalline Na_2_CO_3_ with other components in the sample (such as SiO_2_ and Al_2_O_3_), with C and O volatilising as CO_2_.

**Figure 8:**
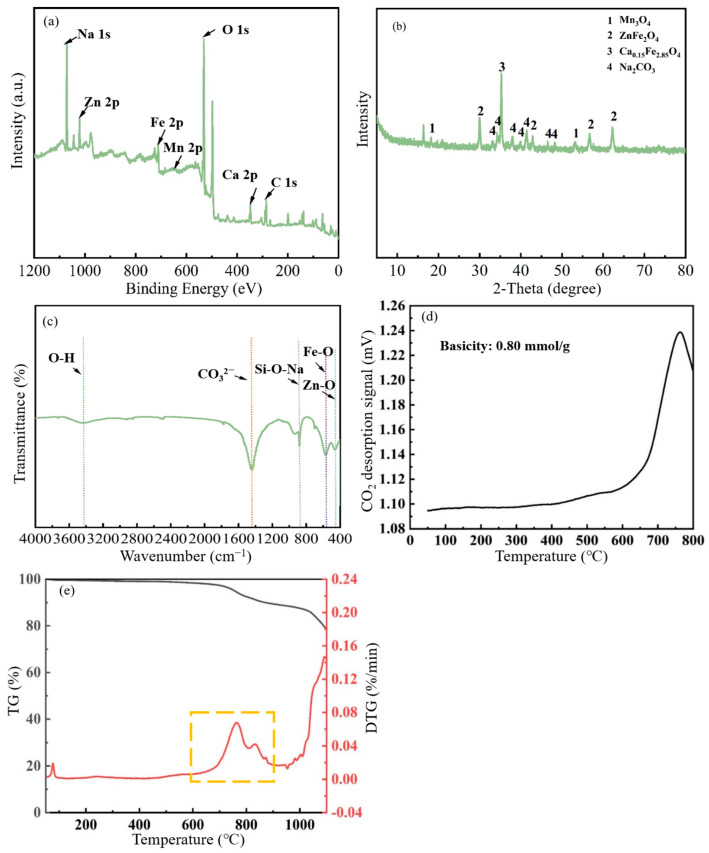
(**a**) XPS survey spectrum, (**b**) XRD pattern, (**c**) FT-IR spectrum, (**d**) CO_2_-TPD profile, and (**e**) TG-DTG curves of (Na_2_CO_3_&(EFD/CH_3_ONa))_600_.

XPS characterisation analysis of (Na_2_CO_3_&(EFD/CH_3_ONa))_600_ (Figure 8a) revealed the relative atomic contents of Na 1s, Zn 2p, Fe 2p, Mn 2p, O 1s, Ca 2p, and C 1s were 13.92%, 2.04%, 5.70%, 0.60%, 51.44%, 2.56%, and 23.75%, respectively. It was noted that the relative content of Na atoms decreased from 16.28% in the original (Na_2_CO_3_&(EFD/CH_3_ONa)) to 13.92%, indicating that the calcination process caused partial sodium loss. The XRD pattern of (Na_2_CO_3_&(EFD/CH_3_ONa))_600_ is shown in Figure 8b. The XRD pattern of calcined Na_2_CO_3_&(EFD/CH_3_ONa) resembled that prior to calcination, but at 2θ = 35.25°, the original Na_2_CO_3_ phase transitions to Ca_0.15_Fe_2.85_O_4_. Comparing this with the crystalline phase detected with EFD/CH_3_ONa (Figure 1A(e)), it could be inferred that following calcination at 600 °C, some Na_2_CO_3_ adhering to Ca_0.15_Fe_2.85_O_4_ detached, allowing the original Ca_0.15_Fe_2.85_O_4_ phase to be detected. This confirmed the aforementioned conjecture. Further analysis of functional groups in the (Na_2_CO_3_&(EFD/CH_3_ONa))_600_ sample via FT-IR revealed, as shown in Figure 8c. The characteristic band of Si-O-Si bonds at 1020–1010 cm^−1^ for the sample Na_2_CO_3_&(EFD/CH_3_ONa) disappeared after calcination at 600 °C, which indicated that the surface Si-O-Si bonds were destroyed. The characteristic spectral band corresponding to CO_3_^2-^ was also weakened. The CO_2_-TPD analysis, shown in Figure 8d, detected a strong basic site in the catalyst between 700 and 800 °C, with a total basicity of 0.80 mmol/L. No other basic sites were identified beyond this [47]. Moreover, the corresponding TG-DTG analysis (Figure 8e) indicated substantial mass loss commencing at 600 °C, potentially attributable to Na_2_CO_3_ decomposition. This finding corresponded to the analytical results from XRD and TEM-EDS.

### 2.4. Analysis of Heavy Metals in Products

Following the 14th catalytic cycle (reaction temperature of 65 °C, reaction time of 2 h, catalyst amount of 7 wt%, and alcohol/oil molar ratio of 15/1), the resulting product was filtered using a 0.22 μm filter head and dried in an oven at 75 °C. The upper layer of biodiesel and the lower layer of glycerol were collected separately for ICP-OES analysis. The concentrations of As, Cd, Cr, Fe, Na, Pb and Sr detected in the biodiesel were 0.30, 0.00, 0.36, 0.87, 0.29, 0.12, and 0.01 mg/L, respectively. In glycerol, the concentrations of As, Cd, Cr, Fe, Na, Pb, and Sr were 0.19, 0.00, 0.45, 2.95, 267.60, 0.69, and 0.04 mg/L, respectively. Only a small quantity of active components (Fe and Na) was leached, with the leached components predominantly deposited in the glycerol. Furthermore, the levels of harmful metals in both the biodiesel and glycerol were below 1.00 mg/L, complying with China’s B5 diesel national standard (95% petroleum diesel and 5% biodiesel) (total metal content < 5 mg/L; GB 25199-2017B5) [48]. This indicated that the solid-waste-based catalyst employed in this study possesses a certain degree of resistance to leaching, and the biodiesel produced can be used as a conventional fuel.

## 3. Materials and Methods

### 3.1. Experimental Materials and Reagents

The soybean oil used in this study (acid value (AV) = 0.02 mg KOH/g, molecular weight (MW) = 860.49 g/mol, saponification value (SV) = 195.60 mg KOH/g) was purchased from a supermarket in Tangshan City, China. Electric furnace dust (abbreviated: EFD) originates from a steelworks in Tangshan City. The EFD powder was dried in an oven at 105 °C to constant weight and sieved through a 200-mesh screen (<75 μm). The hydrochloric acid (HCl, ≥37%), sulfuric acid (H_2_SO_4_, ≥99.99%), nitric acid (HNO_3_, ≥99.99%), oxalic acid (C_2_H_2_O_4_, ≥99.99%) and ammonia solution (NH_3_·H_2_O, ≥25%) used in the research institute were procured from Tianjin Yongda Chemical Reagents Co., Ltd. (Tianjin, China). Phosphoric acid (H_3_PO_4_, ≥85%), acetic acid (CH_3_COOH, ≥36%), malic acid (C_4_H_6_O_5_, ≥85%), citric acid (C_6_H_8_O_7_, ≥36%), sodium hydroxide (NaOH, ≥96%), potassium hydroxide (KOH, ≥90%), sodium methoxide (CH_3_ONa, ≥97%), potassium methoxide (CH_3_OK, ≥95%), sodium ethoxide (C_2_H_5_ONa, ≥96%), anhydrous sodium carbonate (Na_2_CO_3_, ≥99.99%), potassium carbonate (K_2_CO_3_, ≥99.99%), Lithium carbonate (Li_2_CO_3_, ≥99%), Cadmium carbonate (CdCO_3_, ≥99.99%), Barium carbonate (BaCO_3_, ≥99.99%), sodium carbonate decahydrate (Na_2_CO_3_·10H_2_O, ≥99%), sodium hydrogen carbonate (NaHCO_3_, ≥99%), methanol (CH_3_OH, ≥99.5%), anhydrous ethanol (C_2_H_5_OH, ≥99.5%), dichloromethane (CH_2_Cl_2_, ≥99.9%), methyl heptadecanoate (C_18_H_36_O_2_, ≥99.0%), methyl palmitate (C_17_H_34_O_2_, ≥99.0%), methyl linolenate (C_19_H_32_O_2_, ≥99.5%), methyl oleate (C_19_H_36_O_2_, ≥99.0%), methyl linoleate (C_19_H_34_O_2_, ≥99.0%) and methyl stearate (C_19_H_38_O_2_, ≥99.0%) were procured from Shanghai Aladdin Biochemical Technology Co., Ltd. (Shanghai, China).

### 3.2. Preparation of Catalyst

#### 3.2.1. Preparation of Precursors from Acid/Alkali-Modified Electric Furnace Dust

1 mol/L hydrochloric acid, sulfuric acid, nitric acid, phosphoric acid, sodium hydroxide, potassium hydroxide, ammonia water, acetic acid, oxalic acid, malic acid, citric acid, sodium methoxide, potassium methoxide, and sodium ethoxide solutions were used as modification solutions. 5 g of electric furnace dust powder and 50 mL of 1 mol/L modification solution were placed in a 100 mL serum bottle, which was then sealed and transferred to a thermostatic heating magnetic stirrer (DF-101S, Shanghai Lichen Bangxi Instrument Technology Co., Ltd., Shanghai, China) for magnetic stirring at 70 °C for 60 min. The suspension after reaction was transferred to a 1000 mL sand core suction filter flask equipped with a 0.45 μm filter membrane, and the filter cake was rinsed with deionised water until the pH of the washings was around 7. The washed filter residue was placed in an oven (DGG-9140B, Shanghai Senxin Experimental Instrument Co., Ltd., Shanghai, China) at 105 °C to dry to constant weight, ground, and sieved through a 200-mesh sieve (<75 μm), which was named modified electric furnace dust (EFD/S) (where S represented the type of acid or alkali modifier). Approximately 0.8 g of EFD/S was placed in a tube furnace (SK-ES08143, Tianjin Zhonghuan Experimental Electric Furnace Co., Ltd., Tianjin, China) for calcination at 300–900 °C for 2 h (N_2_ flow rate: 200 mL/min; heating rate: 10 °C/min), and named (EFD/S)_T_ (where T represented the calcination temperature).

#### 3.2.2. Catalyst Preparation by Impregnation Activation of Modified Electric Furnace Dust

A 100 mL serum bottle was charged with 1 g of EFD or EFD/S, 1.2 g of activator (metal salt), and 20 mL of deionised water, which was then transferred to a thermostatic heating magnetic stirrer for magnetic stirring at 75 °C for 2 h. The reacted mixture was transferred to an oven at 105 °C to dry to constant weight, ground, and screened through a 200-mesh screen (<75 μm), which was named M&EFD or M&(EFD/S) (where S represented the type of acid or alkali modifier, and M represented the type of activator). Roughly 0.8 g of M&(EFD/S) was placed in a tube furnace and calcined at 300–900 °C for 2 h. (N_2_ flow rate: 200 mL/min; heating rate: 10 °C/min), and the obtained sample was named (M&(EFD/S))_T_ (where T represented the calcination temperature).

### 3.3. Preparation and Analysis of Biodiesel

7 g of soybean oil, 0.49 g of catalyst (with a catalyst amount of 7 wt%) and 4.8 g of methanol (alcohol/oil molar ratio of 15/1) were placed in a vial, which was then sealed with a rubber–aluminium cap. The vial containing the reactants was transferred to a thermostatic heating magnetic stirrer, followed by magnetic stirring at 65 °C for 2 h. After the reaction, the vial was taken out and allowed to stand for 5 min. The mixture in the vial spontaneously separated into three layers under gravity, namely crude biodiesel, a mixture of methanol and glycerol, and solid catalyst. The catalyst at the bottom layer could be directly used in the next cycle without washing and drying. The crude biodiesel was aspirated with a 10 mL syringe, filtered through a 0.22 μm filter head, and then dried in an oven at 75 °C to constant weight to remove excess methanol. Metal concentrations in biodiesel and glycerol were determined using ICP-OES. The filtered and dried biodiesel was analysed using a gas chromatograph (GC-2014 C, Shimadzu Enterprise Management (China) Co., Ltd., Beijing, China) equipped with an Rtx-Wax capillary column (30 m × Φ 0.25 mm × 0.25 μm). Each sample yield was tested twice, with the average value taken. The analysis conditions were as follows: injection port temperature of 260 °C, chromatographic column temperature of 220 °C, detector temperature of 280 °C, carrier gas flow rate of 1 mL/min, and split ratio of 40:1. Dichloromethane was used as the solvent, and methyl heptadecanoate (HAME, C17:0) was used as the internal standard for quantitative analysis of biodiesel. The yield of biodiesel was calculated based on the mass of standard methyl heptadecanoate, correction factor, and peak area. The calculation Formula (1) is as follows:

$$Y=\frac{\sum \frac{W_{HAME}\times A_{FAME}}{A_{HAME}\times F_{i}}}{W_{C}}$$

where

*Y*—Biodiesel yield, wt%;
*W_HAME_*—HAME weight, g;
*A_FAME_*—Chromatographic peak area of *FAME*, uV min;
*A_HAME_*—Chromatographic peak area of *HAME*, uV min;
*F_i_*—Correction factor;
*W_C_*—The weight of crude biodiesel, g.

The correction factors for methyl palmitate, methyl oleate, methyl stearate, methyl linoleate, methyl linolenate and methyl eicosapentaenoate relative to methyl heptadecanoate were 1.000, 0.919, 0.834, 0.936, 1.055 and 0.970, respectively [49].

### 3.4. Characterisation of Catalysts

The elemental content in the samples was measured using an elemental analyser (Vario EL cube, Elementar Analysensysteme GmbH, Hanau, Germany) and an inductively coupled plasma optical emission spectrometer (ICP-OES, Optima 5300, PerkinElmer Inc., Waltham, MA, USA). The crystalline phase characteristics of the samples were analysed using an X-ray diffractometer (XRD, D8 Advance, Bruker AXS GmbH, Karlsruhe, Germany). The surface functional group composition of the samples was detected within the 400~4000 cm^−1^ range using a Fourier transform infrared spectrometer (FT-IR, Nicolet IS10, Thermo Nicolet Corporation, Madison, WI, USA). Thermogravimetric and differential scanning calorimetric analysis of the samples was conducted using a thermogravimetric analyser (TG-DTG, NETZSCH STA449 F5/F3 Jupiter, Netzsch-Gerätebau GmbH, Selb, Germany) within the temperature range of 25–900 °C. Surface alkalinity was determined using a chemical adsorption analyser (CO_2_-TPD, AutoChem II 2920, Micromeritics Instrument Co., Ltd., Norcross, GA, USA). The specific surface area and pore size of the samples were determined using a physical adsorption analyser (ASAP 2460, Micromeritics Instrument Co., Ltd., Northcross, GA, USA) by the Brunauer–Emmett–Teller (BET) method. Field-emission scanning electron microscope equipped with an energy-dispersive spectrometer (SEM-EDS, SU8020, Hitachi, Tokyo, Japan) and high-resolution transmission electron microscope–energy-dispersive spectrometer (HRTEM-EDS, FEI Tecnai G2 F30, Thermo Nicolet Corporation, Madison, WI, USA) were used to characterise the surface morphology and elemental composition of the samples. The saturation magnetisation of the samples was measured using a vibrating sample magnetometer (VSM, SQUID-VSM, MPMS3, Quantum Design International, San Diego, CA, USA). The surface elemental composition and chemical environment of the samples were analysed using an X-ray photoelectron spectrometer (XPS, Thermo escalab 250XI, Thermo Fisher Scientific (China) Co., Ltd., Shanghai, China).

## 4. Conclusions

Overall, the current study synthesised a novel sodium carbonate-supported, sodium methoxide-modified, electric furnace dust-based magnetic multiphase catalyst (Na_2_CO_3_&(EFD/CH_3_ONa)). When subjected to reaction temperature of 65 °C, reaction duration of 2 h, 7% catalyst loading, and alcohol/oil molar ratio of 15/1, the catalyst realised a 100.00 wt% yield of biodiesel. The catalyst demonstrated good activity and stability, exhibiting yields exceeding 93.44 wt% over 14 recycling cycles. Characterisation revealed that the active component Na^+^ forms ionic bonds with hydroxyl oxygen atoms on the modified surface (EFD/CH_3_ONa), creating critical active sites essential for the catalyst’s high performance. Furthermore, the catalyst exhibits a saturation magnetisation of 13.57 Am^2^/kg, sufficient for separating (Na_2_CO_3_&(EFD/CH_3_ONa)) from the reaction system using a magnet. The catalytic performance of the calcined catalyst (Na_2_CO_3_&(EFD/CH_3_ONa))_600_ (effective catalytic cycles: 11) exhibited a decline. Characterisation analysis confirmed this was attributable to partial detachment of Na^+^ from the modified surface (EFD/CH_3_ONa) and subsequent agglomeration. In summary, this study not only provided a viable approach for preparing high-performance magnetic solid-waste-based catalysts for efficient, clean biodiesel production but also pioneered a new pathway for developing green, low-carbon, and high-value utilisation processes for electric furnace dust.

## 5. Research Outlook

Reusability is a key characteristic for evaluating catalyst performance. Current catalysts still suffer from issues such as loss of active components and structural instability of the support during repeated cycles. To further broaden the application of catalysts based on solid waste for the production of biodiesel, future work may consider enhancing catalyst activity and stability by incorporating a binder between the active component and the support. Combining DFT calculations with advanced characterisation techniques (such as synchrotron radiation and three-dimensional reconstruction technology), this approach enables the targeted design of catalytic materials and precise regulation of active sites, thereby achieving industrial standards for the efficient, stable, low-cost, and green production of biodiesel.

## Figures and Tables

**Table 1:** Biodiesel production from soybean oil catalysed by M&EFD.

Types of Catalysts	Biodiesel Yield During Catalyst Recycling (wt%)		
	1	2	3
CdCO_3_&EFD	0.8	-	-
BaCO_3_&EFD	0.7	-	-
Li_2_CO_3_&EFD	4.2	-	-
Na_2_CO_3_·10H_2_O&EFD	66.6	-	-
NaHCO_3_&EFD	88.0	-	-
K_2_CO_3_&EFD	92.7	21.6	-
CH_3_ONa&EFD	89.8	22.4	24.5
C_2_H_5_ONa&EFD	84.3	74.6	40.5
CH_3_OK&EFD	94.4	67.4	64.0
Na_2_CO_3_&EFD	85.6	88.6	47.6

Standard deviation: <5%.

**Table 2:** Biodiesel production from soybean oil catalysed by Na_2_CO_3_&(EFD/S).

Types of Catalysts	Biodiesel Yield During Catalyst Recycling (wt%)														
	1~6	2	3	4	5	6	7	8	9	10	11	12	13	14	15
Na_2_CO_3_&(EFD/HCl)	100.0	100.0	100.0	100.0	93.0	99.8	49.4	18.7	-	-	-	-	-	-	-
Na_2_CO_3_&(EFD/H_2_SO_4_)	100.0	100.0	100.0	100.0	95.1	100.0	87.5	65.8	42.4	-	-	-	-	-	-
Na_2_CO_3_&(EFD/HNO_3_)	100.0	100.0	98.8	100.0	100.0	91.8	75.1	38.9	-	-	-	-	-	-	-
Na_2_CO_3_&(EFD/H_3_PO_4_)	100.0	100.0	90.7	95.9	90.7	90.8	88.0	82.7	70.2	-	-	-	-	-	-
Na_2_CO_3_&(EFD/NaOH)	100.0	100.0	100.0	100.0	100.0	88.5	53.7	23.5	4.5	-	-	-	-	-	-
Na_2_CO_3_&(EFD/KOH)	100.0	100.0	100.0	100.0	100.0	100.0	100.0	96.4	89.2	33.2	-	-	-	-	-
Na_2_CO_3_&(EFD/NH_3_·H_2_O)	100.0	100.0	100.0	100.0	100.0	100.0	100.0	100.0	98.4	66.0	-	-	-	-	-
Na_2_CO_3_&(EFD/CH_3_COOH)	100.0	100.0	100.0	100.0	100.0	100.0	96.8	96.6	95.3	90.4	60.0	-	-	-	-
Na_2_CO_3_&(EFD/H_2_C_2_O_4_)	100.0	100.0	100.0	100.0	92.9	82.4	51.5	11.2	1.8	-	-	-	-	-	-
Na_2_CO_3_&(EFD/C_4_H_6_O_5_)	100.0	100.0	100.0	100.0	100.0	100.0	99.5	100.0	50.7	-	-	-	-	-	-
Na_2_CO_3_&(EFD/C_6_H_8_O_7_)	100.0	100.0	100.0	100.0	100.0	96.7	100.0	100.0	60.1	-	-	-	-		-
Na_2_CO_3_&(EFD/CH_3_ONa)	100.0	100.0	100.0	100.0	100.0	100.0	100.0	100.0	100.0	100.0	100.0	97.8	98.1	93.4	87.6
Na_2_CO_3_&(EFD/CH_3_OK)	100.0	100.0	100.0	100.0	100.0	98.7	97.9	98.4	100.0	98.7	100.0	92.9	82.8	74.0	-
Na_2_CO_3_&(EFD/C_2_H_5_ONa)	100.0	100.0	100.0	100.0	96.3	99.7	12.1	-	-	-	-	-	-	-	-

Standard deviation: <5%.

**Table 3:** Effect of different calcination temperatures on the catalytic performance of Na_2_CO_3_&(EFD/CH_3_ONa).

Calcination Temperature (°C)	Biodiesel Yield During Catalyst Recycling (wt%)							
	1~6	7	8	9	10	11	12	13
300	98.8~98.7	80.9	-	-	-	-	-	-
400	99.7~61.0	-	-	-	-	-	-	-
500	99.9~98.5	98.2	98.5	97.3	66.3	48.3	-	-
600	99.8~98.3	98.2	98.2	97.3	97.6	95.6	69.8	67.4
700	99.8~98.5	98.0	97.2	98.3	97.7	91.3	58.6	17.0
800	99.9~98.6	98.3	98.4	97.6	97.0	90.2	51.4	21.6
900	99.7~98.8	98.0	97.6	97.5	96.2	90.1	50.9	42.7

Standard deviation: <5%.

## Data Availability

The original contributions presented in this study are included in the article.
